# Piezoelectric Transducer-Based Diagnostic System for Composite Structure Health Monitoring

**DOI:** 10.3390/s21010253

**Published:** 2021-01-02

**Authors:** Egidijus Dragašius, Darius Eidukynas, Vytautas Jūrėnas, Darius Mažeika, Mantas Galdikas, Arkadiusz Mystkowski, Joanna Mystkowska

**Affiliations:** 1 Faculty of Mechanical Engineering and Design, Kaunas University of Technology, Studentu str., 56-321 Kaunas, Lithuania; egidijus.dragasius@ktu.lt (E.D.); darius.eidukynas@ktu.lt (D.E.); darius.mazeika@ktu.lt (D.M.); mantas.galdikas@techvitas.lt (M.G.); 2 Institute of Mechatronics, Kaunas University of Technology, Studentu str., 56-005 Kaunas, Lithuania; vytautas.jurenas@ktu.lt; 3 Faculty of Electrical Engineering, Bialystok University of Technology, Wiejska 45D, 15-351 Bialystok, Poland; a.mystkowski@pb.edu.pl; 4 Faculty of Mechanical Engineering, Bialystok University of Technology, Wiejska 45C, 15-351 Bialystok, Poland

**Keywords:** diagnostic system, health monitoring system, defect detection, composite material, piezoelectric sensor, composite defect, mechanical impact

## Abstract

This paper focuses on the investigation of the diagnostic system for health monitoring and defects, detecting in composite structures using a piezoelectric sensor. A major overview of structural defects in composite materials that have an influence on product performance as well as material strength is presented. Particularly, the proposed diagnostic (health monitoring) system enables to monitor the composite material plate defects during the exploitation in real-time. The investigated health monitoring system can indicate the material structure defects when the periodic test input signal is provided to excite the plate. Especially, the diagnostic system is useful when the defect placement is hard to be identified. In this work, several various numerical and experimental studies were carried out. Particularly, during the first study, the piezoelectric transducer was used to produce mechanical excitation to the composite plate when the impact response is measured with another piezoelectric sensor. The second study focuses on the defect identification algorithms of the raw hologram data consisting of the recorded oscillation modes of the affected composite plate. The main paper results obtained in both studies enable us to determine whether the composite material is characterized by mechanical defects occurring during the response to the periodic excitation. In case of damage, the observed response amplitude was decreased by 70%. Finally, using the time-domain experimental results, the frequency response functions (FRFs) are applied to damage detection assessment and to obtain extra damage information.

## 1. Introduction

The development of the industry encouraged the evolvement of the actually used materials. The number of market products based on composite materials is still increasing since they were introduced. Composite materials have different properties because they can be made using a variety of ingredient materials [1,2]. Furthermore, these materials are characterized by a beneficial ratio between the structure mass and strength [3]. Therefore, composite materials can be applied to replace metals and to reduce the total weight of products without decreasing their high strength. Additionally, composite materials offer corrosion-resistant solutions together with cost reduction, high durability, and low maintenance requirements in a wide range of products. Composite materials can be applied in many fields, starting with the production of a simple holder to a musical instrument, ending with aeronautical components. However, if composite materials are used to produce critical parts, effective diagnostic methods and tools are necessary as smart sensors for solidification detection in composite materials [4]. Diagnostic tools can be used during both the production and exploitation of a composite material [5].

**Figure 1:**
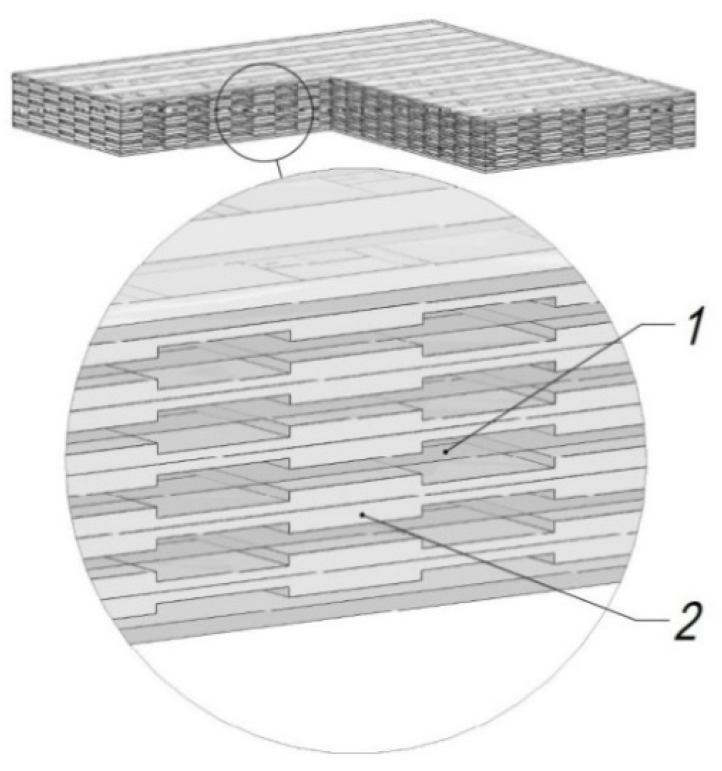
Composite material: 1—binding agent; 2—reinforcement (carbon or glass fibers).

Composite structures are composed of a few different compounds. Some of them are used as reinforcement, others as binders (polymer matrix) (see Figure 1) [5] or fillers (various embedded particles). To produce a composite material, a variety of textiles, fibers, recyclables, wood, metal, etc., can be applied. Using these materials together with layers of the bonding material (i.e., glue, resins, cement, lime), composite material is manufactured [6]. The formed composite product made with different combinations of materials enables to achieve lighter weight and a beneficial ratio between the structure mass and strength in comparison with products based on traditional metal-based ingredients. These properties are determined by the material composition and their formation, not just by qualities of individual components. Other properties which determine high strength of the composite material are the absence of defects and good adhesion between the binding system and the reinforcement [7].

Composite materials’ defects (crack, delamination, etc.) may arise as a result of the technological properties of the manufacturing process, as well as mechanical impact excessing stress allowance during the exploitation [8,9].

The defects in the technological process may occur in a variety of circumstances, but in most cases, are due to the lack of consideration of the sufficient technological conditions of production or as a result of improper selection of composite material structure [10,11]. Some of them are caused by the binding agent or reinforcement material properties, while others by the technological regimes during the production process [12,13]. Several causes of defects may be pointed out.

### 1.1. Defects Due to the Volume Change of Binding Agent during the Curing Process

**Figure 2:**
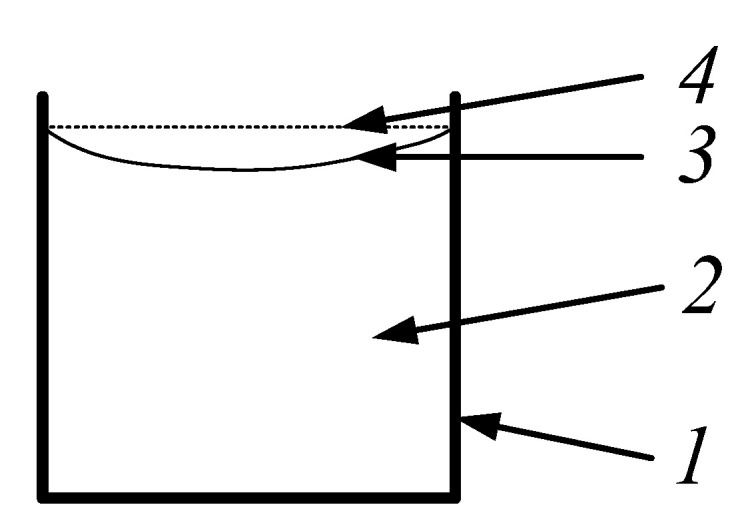
Binder volume change during curing: 1—utensil; 2—stagnant binder; 3—top surface of stagnant binder; 4—top surface of liquid binder.

The binding agent is responsible for polymerization shrinkage of the polymer composite. As is shown in Figure 2, the polymerization shrinkage decreases with the material volume increase. It is caused by a chemical reaction that takes place during the curing process through some part of the liquid binder which turns into a gas. After liquid binding agent evaporation, the total material volume decreased. Based on the research published elsewhere [14,15,16], it was found that the change of volume is directly dependent on the amount of used binder. Even in small quantities of binding agent, the shrinkage can have a significant influence on the properties of composite materials.

### 1.2. Defects Due to the Formation of Gas Bubbles

During the chemical reaction of the curing process, the small part of a liquid binding agent turns into gas, and this gas goes out. As a result, the formation of gas bubbles can be observed. For example, to minimize composite material manufacturing time, the binder or reinforcement material attempts to dry up in the shortest time possible. In this case, when the binder is heated, some of its elements evaporate more intensively than others and increase the probability of forming undesirable gas bubbles. Additionally, bubbles can occur due to inappropriate (too fast) binder mixing in the beginning phase of the production process.

### 1.3. Defects Due to the Insufficient Binder Penetration into the Reinforcement or Insufficient Binder Adhesion to the Reinforcement

These defects may arise from improper binder consistency, temperature and insufficient impregnation time. If the binding agent is not completely absorbed or the gas bubbles are formed, then these composite material areas do not have required properties (i.e., desired strength) [4,6,17]. Due to all of these defects, the composite material does not gain adequate characteristics, which reduces the total strength and may lead to further defects observed during the exploitation. To produce composite materials that are durable and free of internal defects, the major properties of the manufacturing process should be monitored. According to the desired parameters, the technological steps of the production process can be adaptively adjusted. In other cases, the diagnostic system can be used to diagnose the manufactured products, to timely notice of defects that occurred during the exploitation.

**Figure 3:**
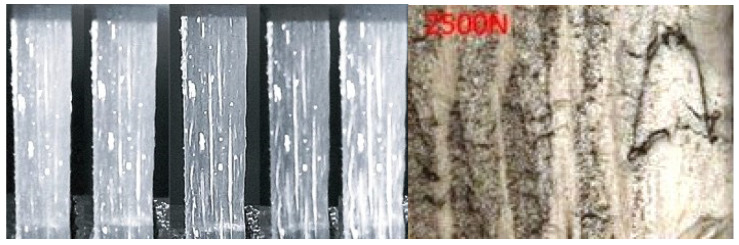

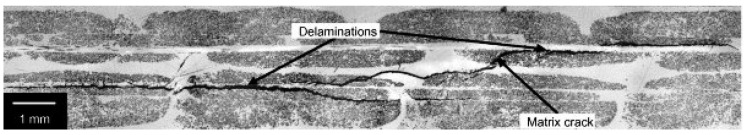
Composite material with defects caused by mechanical impact [12,13,19].

During the composite material operation, due to the mechanical impact, either the binder or reinforcement may be damaged, or delamination may occur. The mechanical impact usually causes micro-cracks or delamination of the material [9,18]. Thus, the resistance of the composite material is reduced in the defect area. Therefore, the mechanical properties of the composite materials depend on the mechanical defects that can increase at any time of the operation (see Figure 3).

In order to detect the mechanical defects of the composite materials in real-time and to protect the composite product from its disintegration, necessary measurements are required to diagnose certain mechanical and thermal parameters of the production and exploitation processes. An overview of applications of piezoelectric sensors for composite defect detection points out that they are widely used due to their unique sensing ad actuating properties [20], both for the detection of homogeneous materials and complex composites. In the work of Jiang et al. [21], stress wave-based active sensing method using piezoceramic transducers (four lead zirconate titanate actuators, where one generates stress waves, and the other three were detectors of wave responses) were used to detect longitudinal grouting quality of the prestressed curved tendon ducts. Another solution [22] is a multi-element sensor including electrical resistivity probes, selective electrodes, and a steel corrosion monitoring system, which enables the real-time and non-destructive monitoring selected parameters. Chen and Chen [23] tested damage detection of nano-SiO_2_ concrete-filled glass fiber reinforced polymer (GFRP) tube column using piezoceramic transducers. They concluded also that this technique can monitor the health status of the structure in real-time and produce early warnings of potential risks. Thus, structural health monitoring of composite structures with use of embedded piezoelectric sensors is of great significance in recent research of composites defects [24,25,26,27,28,29]. The piezo-based macro-fiber composite magnets were applied as the self-powered rotor vibration monitoring system in the active magnetic bearing application [30]. In this application, the piezo-transducers were used both as the energy harvesting and rotor vibration sensing.

The diagnostic systems described in the literature can be divided into implantable and not implantable [4,6,11]:

- implantable diagnostic tools are integrated into the composite material during the manufacturing process and are not considered in this paper;
- not implantable diagnostic tools are not integrated into the composite material, and the detection of the defects is performed by scanning the composite material or by attaching certain sensors to the surface during exploitation.

## 2. Simulation Research of Defect Determination in Composite Material

**Figure 4:**
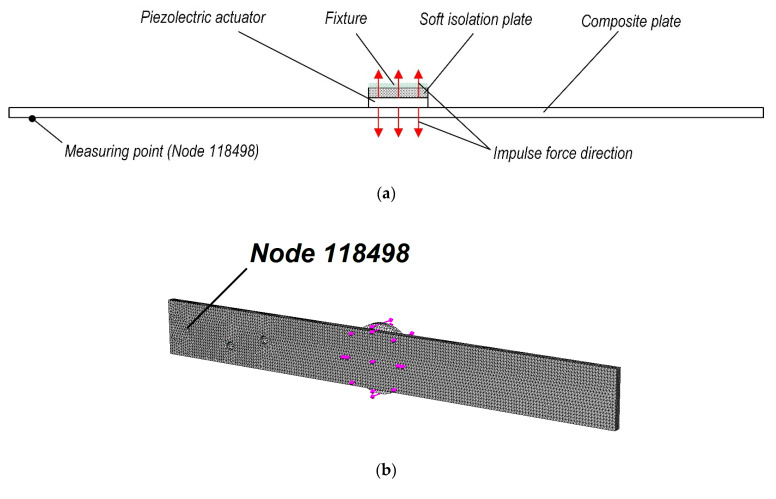

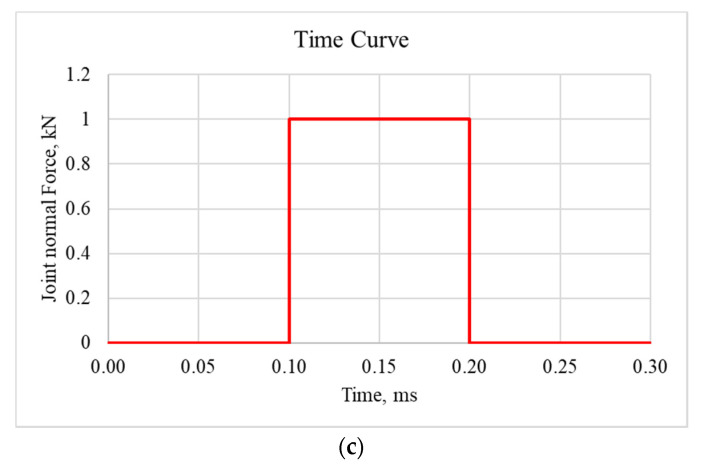
Modeling of the composite plate: (**a**) Schematic representation of boundary conditions used in simulation; (**b**) Computational model with boundary conditions; (**c**) Time response of the excitation impulse force.

In this section, the analytical research and simulation results of the defect determination in the composite material are presented. By using the SolidWorks 2019 nonlinear simulation software, a computational finite-element model of the composite plate was created. Composite plate, in which its properties and geometric characteristics are presented in Table 1, was excited with impulse force (parameters presented in Figure 4c), added on opposite sides of a piezoelectric transducer (actuator) (Figure 4a), and its displacement response was measured at nodal point no. 118498. Notice, that both excitation and measuring points correspond to the centers of excitation and measuring transducers, respectively, used in experimental research. Contact between all parts—composite plate, piezoelectric actuator, and soft isolation layer was set as bonded. All this structure was fixed on the soft isolating material layer’s (parameters presented in Table 1) face, as it was done in experimental research. Schematic representation of boundary conditions is presented in Figure 4a. The application of piezoelectric sensors in damages or different type of defect detection was used by other authors [20,21,31]; as for a structural health monitoring (SHM) system, this method is of great significance [32].

The geometric dimensions and properties of the elements used for the numerical calculations are collected in Table 1.

Figure 4b shows the finite-element numerical model with boundary conditions. This model consists of 76,754 finite elements with 119,280 nodal points when the plate has two defects. The calculations’ impulse excitation time was equal to 0.1 ms. The calculations were carried out in three cases:

(i) the plate has no defects;
(ii) the plate has one defect—Ø2.5 mm hole;
(iii) the plate has two defects—Ø2.5 mm hole each.

Every single defect is modeled as Ø2.5 mm hole. The placements and dimensions of the defects in the pate’s numerical model locations correspond in experimental research, presented in Section 3.

The global damping matrix during the numerical calculation is given by:

(1) a=α[M]+β[K]

where **M** and ***K*** is the mass and stiffness matrix, and α = 4, β = 6 × 10^−6^ are the mass and stiffness coefficients, respectively.

Finite Element Model (FEM) numerical simulation data are collected and presented in Table 2.

**Figure 5:**
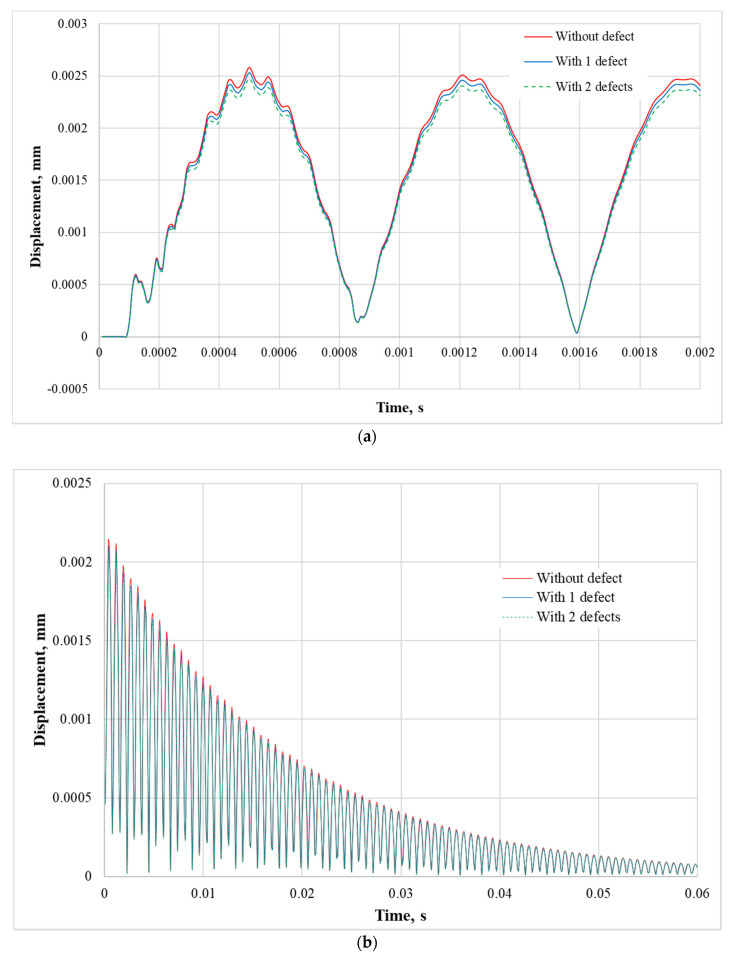
Displacement response of the composite plate displacement response (of nodal point no. 118498) which consists of 119280 nodal points to the impact force; red, blue and green curves present the composite plate without defect, with one defect and with two defects, respectively: (**a**) Simulation time of 0.002 s; (**b**) Simulation time of 0.06 s.

Figure 5a presents modeling results—the composite plate displacement response (of nodal point no. 118498) to the impulse force obtained for the simulation time of 0.002 s when the nodal point is located on the edge of the plate. Similarly, the displacement response of this nodal point for the time of 0.06 s is presented in Figure 5b. Notice, that during the experimental tests, the piezoelectric transducer (piezoelectric sensor) is also attached at this nodal point.

As is shown in Figure 5a, the composite plate (with a total of 119,280 nodal points) displacement at the edge is higher for not damaged material (red curve) compared to the composite plates with one or two defects (blue and green curves). These results show that vibration amplitude obtained at the edge of the composite plate is increasing with the increasing number of the defects. Thus, in the damaged composite material, some part of the vibration energy is dissipated in the composite material defects. Figure 5b shows the composite plate displacements, where the vibration amplitude goes to zero asymptotically. In the case of no defects, the system damping is enough to spread out the composite plate vibrations within 0.06 s after impact force excitation. However, the composite plate with defects has the internal damping and the total time of the transient displacement equals 0.055 s.

## 3. Experimental Research of Defect Determination in the Composite Structure

This section presents experimental results which are carried out to verify the numerical study and simulation methods. For experimental research, the composite material made of 10 fiberglass layers (reinforcement) and with the epoxy resin L285 + H285 (binder and hardener) was used. This combination of the epoxy resin H285 and hardener L285 (developed by MGS Kunstharzprodukte GmbH, Stuttgart, Germany) is used, i.e., for the production of gliders, ships and wind power components. In order to confirm the numerical studies given in the previous section, the test rig was designed according to the conditions of the numerical study. The experimental setup consists of the composite plate, one disc-shaped piezoelectric transducer (actuator, for exciting the structure) in holographic tests (Section 3.1), two disc-shaped piezoelectric transducers (one for exciting the structure and another for vibration response measurement) in impact response tests (Section 3.2), and a soft isolation layer, attached between piezoelectric transducer (actuator), intended for exciting the structure and aluminum base. Aluminum base is used for fixing this structure so that the composite plate would be positioned perpendicular to the holography camera. Notice, that piezoelectric transducer used to excite the composite structure has a diameter of 20 mm while the width of the structure is 16.5 mm. However, the disc-shaped piezoelectric transducer is attached in such a way that symmetry of the structure is ensured.

The experimental research is carried out in three phases. Notice, that defects in (ii) and (iii) were created in the same plate, used in phase I:

(i) the composite plate has no defects;
(ii) the composite plate has one defect;
(iii) the composite plate has two defects.

**Figure 6:**
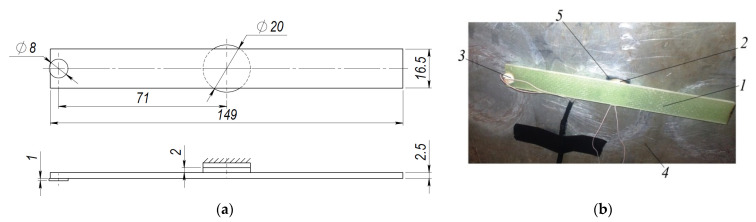
Test setup of the composite plate without defect: (**a**) Dimensions of the composite plate; (**b**) View of the test rig: 1—composite plate, 2—disc-shaped piezoelectric transducer (as the actuator) for exciting the structure, 3—disc-shaped piezoelectric transducer for plate vibration measurement, 4—aluminum base, 5—soft material isolation layer between piezoelectric actuator and aluminum base.

**Figure 7:**
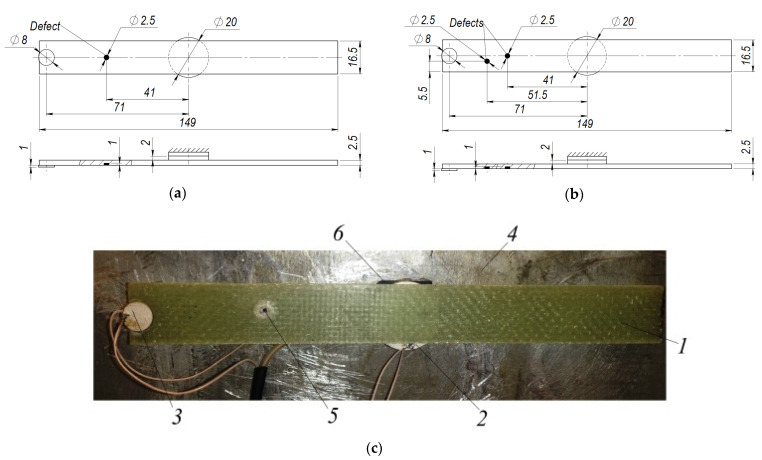
Details of the composite plate with defect: (**a**) Dimensions of the composite plate with one defect; (**b**) Dimensions of the composite plate with two defects; (**c**) View of the composite plate with one defect: 1—composite plate, 2—disc-shaped piezoelectric transducer (as the actuator) for exciting the structure, 3—piezoelectric transducer (as the sensor) for plate vibration measurement, 4—aluminum base, 5—defect, 6—soft material isolation layer between piezoelectric actuator and aluminum plate.

**Figure 8:**
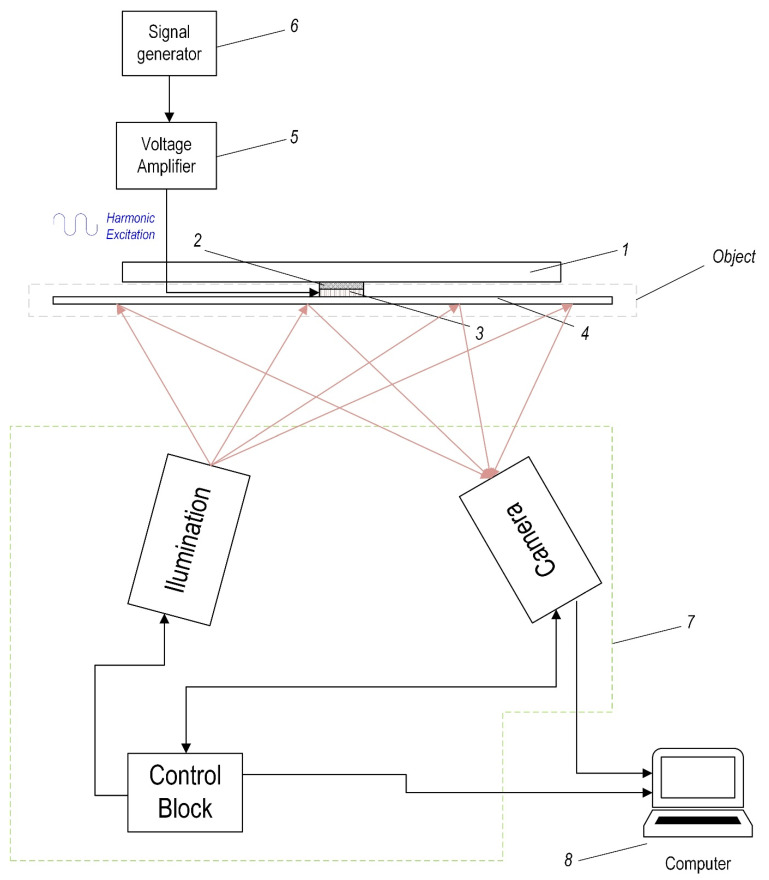
Scheme of holographic experimental set-up: 1—aluminum base (perpendicular to illumination and camera), 2—soft isolation layer, 3—piezoelectric transducer–actuator (for excitation), 4—composite plate, 5—voltage amplifier EPA 104, 6—signal generator Tabor WW5064, 7—holographic system PRISM, 8–PC.

**Figure 9:**
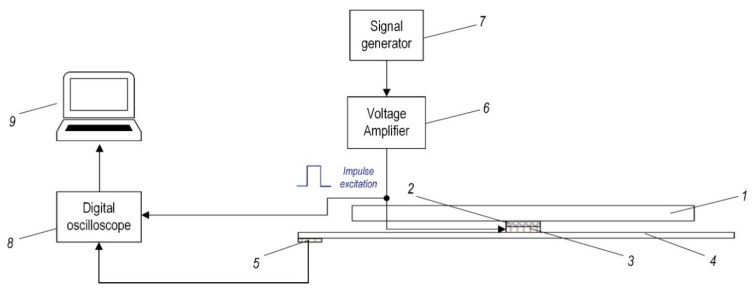
Scheme of experimental set-up during the mechanical impact force investigations: 1—aluminum base, 2—soft isolation layer, 3—piezoelectric transducer–actuator (for excitation), 4—composite plate, 5—piezoelectric transducer–sensor, 6—voltage amplifier EPA 104, 7—signal generator Agilent 33220A, 8—digital oscilloscope PicoScope 6407, 9–PC.

Properties of the investigated composite plate are given in Table 1. The geometrical dimensions of the composite plate and view of the diagnostic system are presented in Figure 6, Figure 7, Figure 8 and Figure 9. For every one experimental phase (i)–(iii), two types of experimental tests were performed:

(i) holographic image (Section 3.1);
(ii) mechanical impact response (Section 3.2).

The plate with one defect (Ø2.5 mm hole) and two defects (Ø2.5 mm hole each) with their locations is given in Figure 7.

### 3.1. Defect Monitoring Using the Holographic Image Method

Figure 8 shows the scheme of the holographic research experimental setup. The experimental setup consists of the composite plate, disc-shaped piezoelectric transducer for exciting the composite structure, and soft isolation layer, attached between piezoelectric actuator and the aluminum base. Aluminum base is used for fixing this structure so that the composite plate would be positioned perpendicular to holography camera.

Holography is a method for the visual representation of dynamic processes based on diffraction and interference of coherent lightwave. The hologram contains information about surface vibration and deformation with no contact with the object. In this research, the HYTEC PRISM system with measurement sensitivity <20 nm and measurement range >0.1 mm, which, in general, is a two-beam speckle pattern interferometer, was used. During this experimental research, harmonic excitation signal from the generator with frequency varying in range 0–50 kHz is applied to piezoelectric actuator (pos. 2, Figure 8) and thus, vibration energy is generated. This energy is transferred to the composite plate and its surface vibrations are measured using holography method.

As is described above, the experimental research is carried out in three phases: First, when the plate has no defects; second, when the plate has one defect and third, when the plate has two defects. In particular:

- during the first phase, the holographic tests of the composite plate without defects are performed where disc-shaped piezoelectric transducer *3* attached to the composite plate *4* was used as the vibration generator (see Figure 8);
- during the second phase, the mechanical impact tests of the composite plate with one defect are performed. Excitation and measuring conditions are the same as mentioned above. The composite plate defect was made by inserting a steel ball with a press in which reinforcement and binder were damaged;
- during the third phase, similar experimental tests are carried out for the composite plate with two defects. Excitation and measuring conditions are the same as mentioned above. The locations of the mechanical defects are shown in Figure 7.

The holographic studies have shown that vibration excitation of the composite plate with certain resonant frequency enabled to observe changes in the holographic images according to the number of the defects in composite material. These changes depend on the composite plate vibration modes. In this way, the defects in the composite material can be detected by revealing the differences in the vibration modes obtained at the same excitation frequencies for the three test phases (i)–(iii). Table 3 shows the holographic images of the composite material plate obtained for the resonant frequencies. As it is seen from these results, in all resonant frequencies, differences between plate without defect and with one and two defects, respectively, are obtained. The white color of holographic images shows moving points of surfaces while black—stationary. In comparison, holographic images of the same resonant frequencies, with and without defects, show that defect changes moving surface area—usually moving area increases, i.e., changes its shape and thus, asymmetry of the vibrating plate is obtained. However, the holographic image method of defect detection in the composite plate is difficult to realize in practice. For this reason, the mechanical impact method is proposed in the next Section 3.2.

### 3.2. Experimental Research of the Mechanical Impact Response

This section presents the experimental validation of the simulation results of the frequency of the composite plate as the response to the mechanical impact tests. Scheme of the experimental set-up is shown in Figure 9. The experimental study of the mechanical impact test is carried out in three phases: In the first phase, the composite plate has no defects; at the second phase, the composite plate has one defect and at the third phase, the composite plate has two defects. Parameters of impulse excitation, generated with the signal generator, are the same as previously used for simulation; see Figure 4c.

In the first phase (Figure 6), the test stand consists of the composite plate *1* without defects and disc-shaped piezoelectric transducer *2* (PZT-5) for impulse excitation acting as the actuator attached to the center of the composite plate. In this research, another disc-shaped piezoelectric transducer *5* (PZT-5) acting as the sensor is attached at the edge of the composite material plate. This transducer converts the vibration energy to the electric signal which is recorded by the digital oscilloscope (PicoScope-6407) for analysis. Since the composite material plate with the piezoelectric actuators should not be attached directly to the solid aluminum base *4,* the additional soft material isolation layer is used.

**Figure 10:**
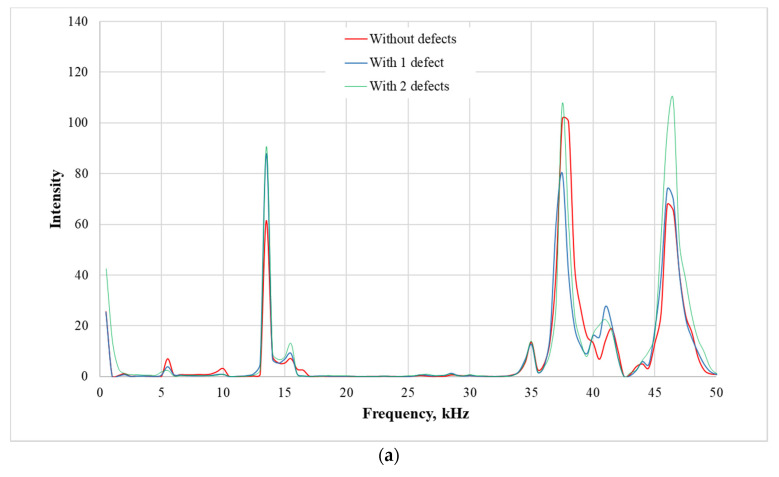

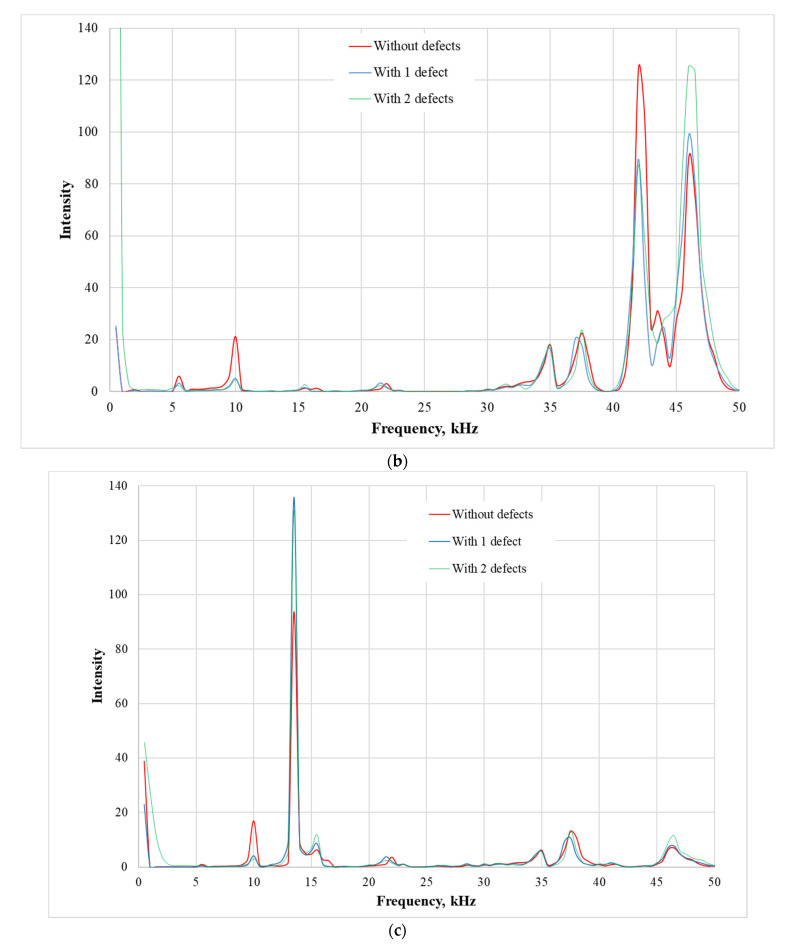
Spectrograms of the composite plate responses to the impact force for the impulse duration time of: (**a**) 9.5 × 10^−5^ s; (**b**) 7.7 × 10^−5^ s; and (**c**) 2.4 × 10^−5^ s, respectively.

During experimental research, the vibration measurements are performed and the piezoelectric transducer *3* was used as the mechanical impact generator (see Figure 9). The impact response energy of the composite plate is transferred to the piezoelectric transducer *5.* This transducer converted the mechanical vibrations’ energy to the electrical signal recorded by the digital oscilloscope. Next, comparison and analysis of the vibration responses of the damaged and not damaged composite plate are carried out. The spectrogram analysis was performed using MATLAB^®^ software. The obtained results are presented in Figure 10.

The spectrograms of the composite plate responses to the mechanical impact input show that the composite material plate without defect and the composite material plate with a defect give different forms of the signals according to the frequency and amplitude of the resonances. These differences in the signals are essential to the identification of the defect in the composite plate. The excitation frequency higher than 50 kHz is not informative at all due to environmental noise. Therefore, the spectrograms are analyzed in the frequency range from 1 Hz to 50 kHz. The highest response amplitudes are observed at the given impulse force-time durations of 9.5 × 10^−5^ s, 7.7 × 10^−5^ s and 2.4 × 10^−5^ s, respectively. Moreover, the most accurate results which indicate the composite material conditions are obtained at the frequency of 5.5 kHz, 10 kHz, 13 kHz, 37.5 kHz, 42.5 kHz and 47 kHz. These maximal values of the amplitudes (pk-to-pk) depend on the impact force duration time. In particular, in the spectrograms, the red curve represented the oscillation intensity of the composite material plate without defects; the blue one—with one defect and the green one with two defects. In the presented spectrograms, at the certain vibration frequencies of the composite plate (5.5 kHz, 10 kHz and 47 kHz), different vibration amplitudes are obtained. At the frequency of 5 kHz and 8 kHz, the vibration amplitude of the composite plate with no defect (red curve) is the highest, the vibration amplitude of the composite plate with one defect (blue curve) is decreased by 5% and the vibration amplitude of the composite plate with two defects (green curve) is decreased by 70%. At the frequency of 47 kHz, these results are opposite.

### 3.3. Frequency Response Function-Based Damage Detection

In this section, the frequency response function- (FRF) based algorithm is applied to damage detection, as in work [33]. The FRF data are obtained from the raw measurements of the composite plate responses to the input impact force, when the impulse duration time was 9.5 × 10^−5^ s, as the most indicated one. The major advantage of FRF algorithms is that it gives significant information about the dynamic response of the structure over a frequency range [34]. The FRF is one of the modal analyses, where the calculated modal data are damage evaluable without loss of the system information that can influence on damage assessment. The main obstacle in using FRFs for defect detection is the potentially large size of the FRF data. Therefore, to the effective realization of the damage assessment algorithm, the FRF data have to be reduced without missing a critical issue. Moreover, accordingly, in this work, another method of damage assessment is derived utilizing a so-called “damage index” (DI) technique. The DI is the ratio between FRF data obtained from damaged structure and undamaged structure and therefore provides a useful tool in damage system assessment.

For the system with the input *x* and output *y* signals in the frequency domain, the FRF function is defined as:

(2) $$H=\frac{S_{xy}}{S_{xx}}$$

where $S_{xy}$ and $S_{xx}$ is the crosspower and autopower complex functions.

For given measurements in data vectors of the damage system, the FRF matrix data is collected as:

(3) $$H_{damage}={\left[h_{ij}\left(\omega \right)\right]}_{damage}$$

For the *m* rows of the FRF, the mean value of the jth column of undamaged data set is defined as:

(4) $${\left[\bar{H_{j}}\right]}_{undamage}=\frac{1}{m}\overset{m}{\underset{i=1}{\sum }}h_{ij}\left(\omega \right)$$

The standard deviation of the FRF is defined as:

(5) $$S_{j}^{2}=\frac{1}{m}\overset{m}{\underset{i=1}{\sum }}{\left(h_{ij}\left(\omega \right)-\bar{H_{j}}\right)}_{undamage}^{2}$$

Then, the element of the FRF matrix of damage structure is replaced with:

(6) $${\left[{\tilde{h}}_{ij}\left(\omega \right)\right]}_{damage}=\frac{{\left[h_{ij}\left(\omega \right)\right]}_{damage}-{\left[\bar{H_{j}}\right]}_{undamage}}{S_{j}\sqrt{m}}={\left[\tilde{H}\right]}_{r\times r}$$

For correlation matrix of damage structure ${\left[C\right]}_{rxr}={\left[\tilde{H}\right]}^{T}{}_{r\times m}{\left[\tilde{H}\right]}_{m\times r}$, the singular value decomposition (SVD) can be given as:

(7) $${\left[C\right]}_{rxr}=\left[\Psi_{i}\right]\Lambda {\left[\Psi_{i}\right]}^{T}$$

where Ψ is the eigenvector values of the correlation matrix and Λ is the positive diagonal matrix.

The principal components (PCs) are the eigenvalues associated with the eigenvectors [Ψ] of the original data. The projection of the response of the variation matrix ${\left[{\tilde{H}}_{new}\right]}_{m\times r}$ on the PCs derived from FRFs of the damage system is given as:

(8) $${\left[A\right]}_{m\times r}={\left[{\tilde{H}}_{new}\right]}_{m\times r}{\left[\Psi \right]}_{r\times r}$$

Based on the studies of [34,35], the projection matrix A can be partitioned into sub-systems, where *p* is the significant PCs. This is the main way to reduction of the PCs’ number. Setting those sub-matrices representing (*r–p)* to 0, we obtain a new constructed signal:

(9) $${\left[A\right]}_{m\times p}{\left[\Psi \right]}^{T}{}_{r\times r}={\left[H_{R}\right]}_{damage}$$

In this way, we can calculate the damage index (DI) that is the ratio between FRFs’ data obtained from damaged structure and undamaged structure. The DI for the *k*-th subset of d damage case is defined as:

(10) $${\left[DI_{d}\right]}_{k}={\left[H_{\mathrm{Rd}}\right]}_{k}/{\left[{H_{\mathrm{mean}}}_{undmage}\right]}_{k}$$

where k=[1,2, …,n/r], and ${\left[H_{\mathrm{Rd}}\right]}_{k}$ is the row of damage case d from reconstructed FRFs.

**Figure 11:**
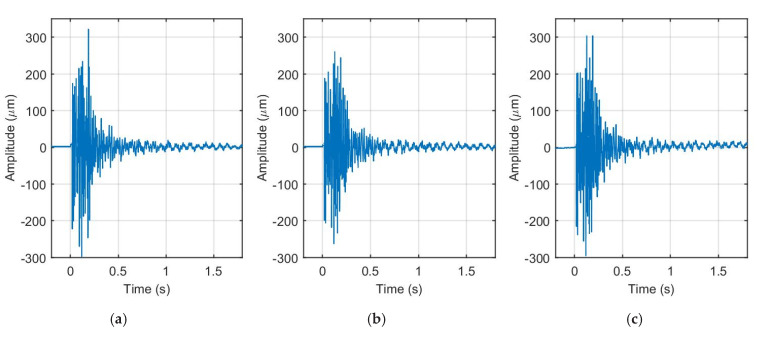
Impact force time responses: (**a**) Without defect; (**b**) One defect; (**c**) Two defects.

**Figure 12:**
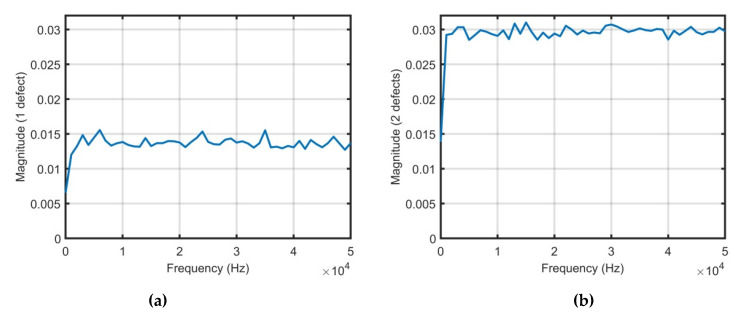
Frequency response functions (FRFs): (**a**) One defect; (**b**) Two defects.

The measured impact force time responses of the undamaged and damaged cases with one or two defects in the composite plate, are presented in Figure 11. The experimental results of the frequency responses of the given cases were given previously in Figure 10. The FRFs for given frequencies from 0 to 5 kHz are calculated and presented in Figure 12.

**Figure 13:**
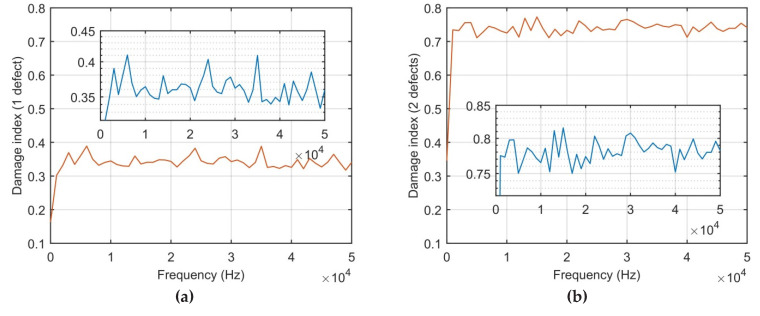
Damage index values: (**a**) One defect; (**b**) Two defects.

The FRFs’ responses given in Figure 12 are obtained from the time-domain responses’ data denoted in Figure 11. The FRFs are calculated only for damaged cases, when one defect and two defects are in the structure, respectively. Note, that in case of the structural defect location problem, the difference between the FRF vectors can be used to assess the damage point placement. The ratio between FRFs’ data obtained from the damaged structure (case 1 and 2) are compared with the undamaged structure data set. The obtained DI is presented in Figure 13.

Based on the results of the DI for case 1 and 2 compared to the undamaged structure, it can be observed that the DI for each damaged case has different states. The composite plate with two defects has approx. two times bigger mean value of the DI in comparison with case 1 (when one defect has occurred). Moreover, the DI values with respect to frequencies show the ratio of the FRFs obtained from damaged structure and undamaged structure. The frequency range of the DIs’ calculations is up to 5 kHz in order to avoid the system noise influence and high order modes that are not informative.

**Figure 14:**
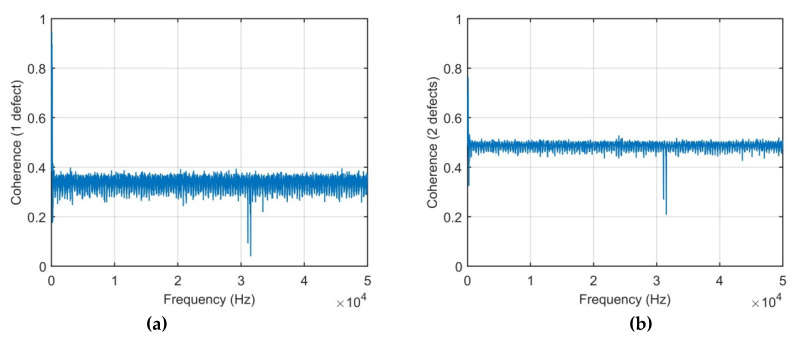
Coherence function: (**a**) One defect; (**b**) Two defects.

To show the relationship between the data set of the damage cases with the undamaged case, the coherence functions are derived and given in Figure 14.

The coherence functions of the case 1 and 2 measure the correlation between the output signal and the input signal at each signal. It is well-known that the maximal value of coherence equals 1 when the output is caused only by the input. In case of the plate structure damage, the coherence is much less than 1 since the damage influences on the structure’s dynamic behavior. The influence of the defect numbers together with input (impact force) signal on the output signal is observed in coherence relationship. These coherence functions are calculated for the impact force-time responses for the given impulse force-time durations of 9.5 × 10^−5^ s.

## 4. Conclusions

Major comments are formulated in the following points:

(i) Simulation and both experiments validated the method of the proposed diagnostic system, which enables to perform the monitoring of the composite materials during the operation. In general, during real operation of the structure, it is possible to use only piezoelectric transducer(s) as a sensor(s) while excitation can be generated from shock with mechanical hummer, ambient vibrations, etc.
(ii) The numerical studies have shown that the composite material plate (which consists of 119,280 nodal points) without a defect had higher oscillation amplitude (displacement) than the composite material plate with one or two defects.
(iii) The holographic images of the composite material plate with and without defects obtained for the same excitation frequency have revealed the differences in oscillation modes. Based on these oscillation modes, the defects of the composite material can be identified.
(iv) The spectrograms, that show the increase of the force impulse duration and changes of the spectrogram amplitude, allow determining the existence of the defect in the composite material. In particular, at the frequency of 5 kHz and 8 kHz, the vibration amplitude of the composite plate with no defect is the highest, where the vibration amplitude of the composite plate with one defect is decreased by 5% and the vibration amplitude of the composite plate with two defects is decreased by 70%.
(v) Identification of the composite material defect is possible by using one piezoelectric transducer as actuator and the second one as the sensor when the defect is located between these transducers.
(vi) Choosing the proper time duration of the impulse mechanical impact is essential in indicating the diagnostic information of composite material.
(vii) Experimental modal analysis was performed using FRFs, which are a useful tool for the system damage indication.
(viii) The DI provides the ratio between FRFs’ data obtained from damaged structure and undamaged structure.
(ix) It is well known that damage detection in composite structures is difficult due to the anisotropy of the material, fiber conductivity and the isolative properties of the matrix. The frequency response methods are reliable for detecting the damage in a simple composite structure, however, the important information about damage location and orientation were lost using this method since these combinations of variables can yield the same responses. This is the main disadvantage of these methods. Another limitation is that these methods appear to be appropriate for monitoring global changes in stiffness, and hence damage, especially when the structures are large. There are also other detection limitations imposed by the resolution and range of the individual sensors chosen, etc., that exist in other sensor-based methods.

## Figures and Tables

**Table 1:** Properties and geometric dimensions of the composite, piezoelectric and soft isolation layer materials.

Parameter	Unit	Value
binding agent (resin + hardener)		L285 + H285
Young’s modulus of composite material	GPa	25
Poisson’s coefficient of composite material		0.2
density of composite material	kg/m^3^	1900
length × width × thickness of composite plate	mm	148.5 × 16.5 × 2.2
diameter × thickness of piezoelectric actuator	mm	ø20 × 2
Young’s modulus of piezoelectric material	GPa	74
Poisson’s ratio of piezoelectric material		0.35
density of piezoelectric material	kg/m^3^	7300
length × width × thickness of soft isolating layer	mm	20 × 10.5 × 2.0
Young’s modulus of soft isolating layer	GPa	7
Poisson’s ratio of soft isolating layer		0.1
density of soft isolating layer	kg/m^3^	1400

**Table 2:** FEM numerical simulation data.

Parameter	Unit	Value
number of finite elements	-	76,754
number of nodal points	-	119,280
excitation shock impulse time	ms	0.1
excitation shock impulse	kN	10

**Table 3:** Resonance holographic images of the composite material plate.

Excitation Frequency, [kHz]	Composite Material Holographic Image
1.8	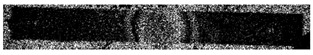 Not damaged composite plate 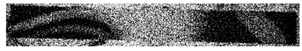 1 defect in composite plate 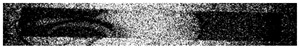 2 defects in composite plate
5.6	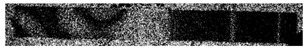 Not damaged composite plate 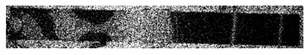 1 defect in composite plate 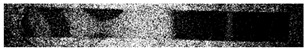 2 defects in composite plate
10.5	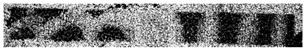 Not damaged composite plate 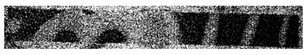 1 defect in composite plate 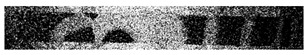 2 defects in composite plate
41.7	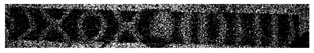 Not damaged composite plate 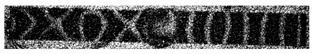 1 defect in composite plate 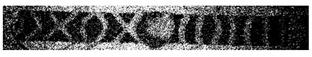 2 defects in composite plate

## Data Availability

Data available in a publicly accessible repository—in this paper.
